# Multimodal therapy including robot‐assisted radical cystoprostatectomy for locally advanced prostate cancer with bladder and ureteral invasion: A case report

**DOI:** 10.1002/iju5.12500

**Published:** 2022-06-29

**Authors:** Masayoshi Okumi, Yuma Kujime, Soichi Matsumura, Hiroaki Kitakaze, Kosuke Nakano, Sachiko Hongo, Iwao Yoshioka, Shingo Takada

**Affiliations:** ^1^ Department of Urology Osaka Police Hospital Osaka Japan

**Keywords:** bladder and ureteral invasion, locally advanced prostate cancer, multimodal therapy, robot‐assisted radical cystoprostatectomy

## Abstract

**Introduction:**

It remains unclear whether robot‐assisted radical cystoprostatectomy for locally advanced prostate cancer represents excessive treatment.

**Case presentation:**

A 58‐year‐old man presented with urinary retention and renal failure. Prostate‐specific antigen level was 38.07 ng/mL and computed tomography scans revealed bilateral hydronephrosis due to prostate enlargement. Prostate biopsy revealed a Gleason score of 5 + 5 adenocarcinoma, and bilateral hydronephrosis persisted even after urethral catheter placement. We diagnosed locally advanced prostate cancer with bladder and ureteral invasion. Percutaneous bilateral nephrostomy was performed, and neoadjuvant hormone therapy was initiated. Four months after the start of hormone therapy, robot‐assisted radical cystoprostatectomy and an intracorporeal ileal conduit were performed, followed by adjuvant radiation therapy for lymph node metastasis. Seven months after the surgery, the patient was free of disease with prostate‐specific antigen level <0.03 ng/mL.

**Conclusion:**

Robot‐assisted radical cystoprostatectomy can be an effective multimodal therapy for locally advanced prostate cancer with bladder and ureteral invasion by locally advanced prostate cancer.

**Keynote message**

As a multimodal therapy, RARC can provide effective radical treatment for patients with bladder and ureteral invasion by LAPC.

**Abbreviations & Acronyms**
eGFR: estimated glomerular filtration ratio
HT: hormone therapy
ICIC: intracorporeal ileal conduit
LAPC: locally advanced prostate cancer
PSA: prostate‐specific antigen
RARC: robot‐assisted radical cystoprostatectomy
RT: radiation therapy
sCr: serum creatinine

## Introduction

Radical cystoprostatectomy can significantly reduce the risk of positive surgical margins of the bladder neck and effectively palliate local symptoms such as perineal pain, hematuria, ureteral obstruction, and voiding dysfunction associated with LAPC.^1^, ^2^, ^3^ However, it may sometimes be an excessive treatment for patients with LAPC. The long‐term oncological outcomes of RARC for LAPC with bladder invasion remain unclear. However, a recent systematic review revealed that RARC may be an alternative to surgery in multimodal therapy for some LAPC patients with bladder invasion,^3^ and may improve urinary symptoms as well as patient quality of life.^4^ Herein, we present a case of LAPC treated with RARC and ICIC, followed by adjuvant RT in a patient with bilateral hydronephrosis due to bladder and ureteral invasion.

## Case presentation

**Fig. 1:**
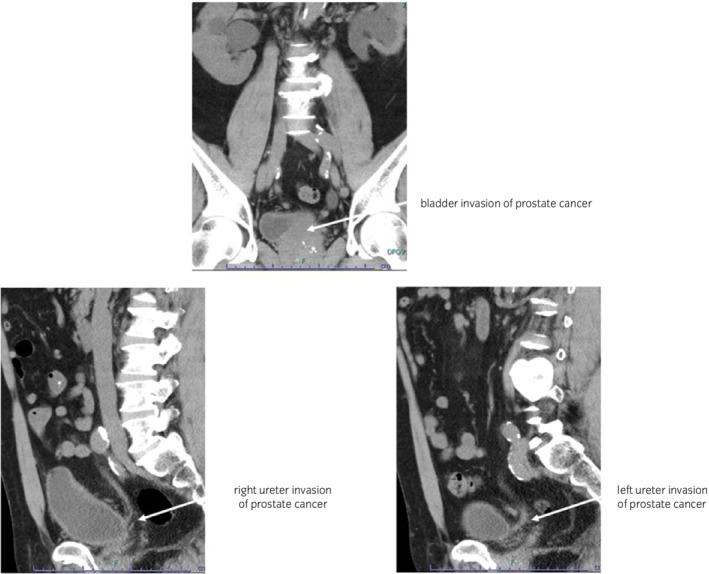
Computed tomography scans show bilateral hydronephrosis due to lower urinary obstruction by bladder and bilateral ureteral invasion of prostate cancer.

A 58‐year‐old man presented with urinary retention and renal failure. Blood tests showed PSA levels of 38.07 ng/mL, sCr levels of 3.94 mg/dL, and eGFR of 13.5 mL/min. Abdominal computed tomography scans revealed bilateral hydronephrosis due to lower urinary obstruction due to prostate enlargement; however, claustrophobia prevented the patient from undergoing magnetic resonance imaging scanning. As his bilateral hydronephrosis persisted and renal function did not improve dramatically even after urethral catheter placement, bladder and ureteral invasion by advanced prostate cancer was strongly suspected (Fig. 1). Prostate biopsy revealed a Gleason score of 5 + 5 adenocarcinoma. We diagnosed LAPC with bilateral hydronephrosis due to bladder and ureteral invasion and planned multimodal therapy, consisting of neoadjuvant HT, radical cystoprostatectomy, and adjuvant RT. Percutaneous bilateral nephrostomy was performed, and neoadjuvant HT was initiated. As upper urinary tract infection occurred every time the nephrostomy catheter exchange, RARC with ICIC which had been approved by the ethical committee of our institution was performed 4 months after the start of HT when PSA levels decreased to below 0.03 ng/mL.

The surgery was performed using the da Vinci Xi Surgical System (Intuitive Surgical, Sunnyvale, CA, USA), with pelvic lymph node dissection of the bilateral obturator, internal to external and common iliac regions, and radical cystoprostatectomy followed by the construction of ICIC. The total console time was 326 min (60 min for lymph node dissection, 69 min for radical cystoprostatectomy, and 142 min for ICIC), and the estimated blood loss was 400 mL. No perioperative complications nor urinary tract infection occurred after urinary diversion, even after nephrostomy catheters were removed.

Histopathology revealed Gleason 5 + 5 adenocarcinoma of the prostate with seminal vesicle, bladder neck, and left lower ureteral invasion without positive surgical margins, but positive lymph node involvement of the bilateral obturator and right iliac regions (8/17), diagnosed as LAPC, EPE1, RM0, ly1, v0, pn1, sv1, ypT4N1.

**Fig. 2:**
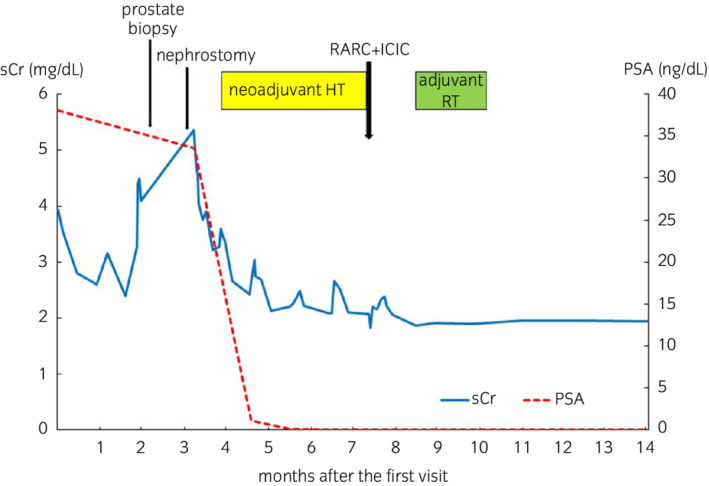
Changes in PSA and sCr level and clinical course during multimodal therapy.

One month after the surgery, renal function has improved with sCr levels of 1.86 mg/dL and eGFR levels of 30.7 mL/min, and the patient underwent adjuvant RT into total pelvic cavity, given the evidence of positive lymph node metastasis. Seven months after the surgery, the patient has maintained PSA levels <0.03 ng/mL and testosterone levels 57 ng/dL without recurrence or metastasis without any additional HT (Fig. 2).

## Discussion

Men with high‐grade prostate cancer and lymph node involvement and who undergo HT are at the greatest risk of progression to castration‐resistant prostate cancer.^5^, ^6^ While RT with androgen deprivation therapy is the current standard of care for the treatment of stage cT4 prostate cancer, radical prostatectomy may be appropriate for some patients undergoing a combination treatment that may include RT and HT.^7^ A study of 1914 men with LAPC revealed that local therapy with radical prostatectomy, RT, or a combination of both improved 5‐year overall survival rates compared to systemic therapy.^8^ However, the definitive role and sequence with which to optimize therapy combinations in this setting remain unknown.

A previous study from a large high‐risk prostate cancer population showed that neoadjuvant HT before surgery significantly decreased cancer‐specific mortality rates, and the beneficial effect of neoadjuvant HT appeared to be mainly driven by the early addition of RT post‐surgery.^9^ The result from a randomized comparative study from the Canadian Uro‐Oncology Group suggested the optimal duration of neoadjuvant HT to occur biochemical and pathological regression of prostate tumor is longer than 3 months.^10^ The investigation of changes in prostate cancer cell proliferation and different durations of neoadjuvant HT showed that the optimal duration of neoadjuvant HT is longer than 3 months.^11^ In our case, the patient underwent neoadjuvant HT for 4 months and radical cystoprostatectomy followed by adjuvant RT 1 month after surgery.

Radical cystoprostatectomy with adjuvant therapy may be recommended for highly selected LAPC patients with bladder invasion, specifically, those with severe urinary symptoms.^3^, ^4^ However, the current guidelines have not yet concluded the optimal therapeutic strategy for LAPC patients with bladder invasion. It has been reported that cystoprostatectomy with immediate adjuvant HT might lead to the achievement of excellent local control for the initial treatment of LAPC patients.^12^ In addition, It has been found that cystoprostatectomy could provide effective and durable palliation with acceptable morbidity as either the initial or salvage treatment for LAPC patients with symptomatic bladder invasion.^2^ While palliative cystoprostatectomy for patients with LAPC provides good short‐term survival outcomes,^13^ long‐term oncological outcomes of radical cystoprostatectomy for LAPC with bladder invasion remain unclear. A prospective randomized control trial to assess oncological outcomes between radical cystoprostatectomy and RT with androgen deprivation therapy against cT4 prostate cancer with bladder invasion is ongoing.^7^

## Conclusion

Neoadjuvant HT + RARC with adjuvant RT provides effective and durable palliation in patients with LAPC. RARC can provide effective treatment with acceptable morbidity for patients with bladder and ureteral invasion by LAPC.

## Author contributions

Masayoshi Okumi: Conceptualization; data curation; formal analysis; investigation; methodology; project administration; visualization; writing – original draft. Yuma Kujime: Data curation. Soichi Matsumura: Data curation. Hiroaki Kitakaze: Data curation. Kosuke Nakano: Data curation. Sachiko Hongo: Data curation. Iwao Yoshioka: Data curation. Shingo Takada: Data curation; methodology; supervision.

## Conflict of interest

The authors declare no conflict of interest.

## Approval of the research protocol by an Institutional Reviewer Board

The study protocol for this research project and treatment has been approved by the ethical committee of our institution (approval number: 1520).

## Informed consent

Consent for publication were obtained from the patient.

## Registry and the Registration No. of the study/trial

Not applicable.
